# 2136 Frequently overlooked challenges of pragmatic trials

**DOI:** 10.1017/cts.2018.213

**Published:** 2018-11-21

**Authors:** Rodger S. Kessler

**Affiliations:** Arizona State University

## Abstract

OBJECTIVES/SPECIFIC AIMS: To review the multiple differences between traditional research design and on the ground pragmatic trials. To review two pragmatic projects, identify core assumptions and to contrast assumptions with the reality of conducting T3 and T4 research. METHODS/STUDY POPULATION: Observational mixed methods multi trial review of large multi site implementations. RESULTS/ANTICIPATED RESULTS: The complexities of implementation on the ground were consistently greater than anticipated and required changing assumptions and research design elements. DISCUSSION/SIGNIFICANCE OF IMPACT: Research findings are tremendously influenced by design and design implementation decisions. Anticipating the scope and breadth of the challenges will assist potential of successful implementation.

